# Rheological Tunability of Perovskite Precursor Solutions: From Spin Coating to Inkjet Printing Process

**DOI:** 10.3390/nano9040582

**Published:** 2019-04-09

**Authors:** Antonella Giuri, Ehab Saleh, Andrea Listorti, Silvia Colella, Aurora Rizzo, Christopher Tuck, Carola Esposito Corcione

**Affiliations:** 1 Dipartimento di Ingegneria dell’Innovazione, Università del Salento, via per Monteroni, km 1, 73100 Lecce, Italy; antonella.giuri@unisalento.it; 2 Istituto di Nanotecnologia CNR-Nanotec, Polo di Nanotecnologia, c/o Campus Ecotekne, via Monteroni, 73100 Lecce, Italy; andrea.listorti@unisalento.it (A.L.); silvia.colella@unisalento.it (S.C.); aurora.rizzo@unisalento.it (A.R.); 3 Centre for Additive Manufacturing, Faculty of Engineering, University of Nottingham, Nottingham NG8 1BB, UK; E.Saleh@leeds.ac.uk (E.S.); Christopher.Tuck@nottingham.ac.uk (C.T.); 4 Future Manufacturing Processes Research Group, School of Mechanical Engineering, University of Leeds, Leeds LS2 9JT, UK; 5 Dipartimento di Matematica e Fisica “E. De Giorgi”, Università del Salento, Via Arnesano snc, 73100 Lecce, Italy

**Keywords:** starch-perovskite inks, rheological tunability, inkjet printing

## Abstract

The high efficiencies (>22%) reached by perovskite-based optoelectronic devices in a very short period, demonstrates the great potential and tunability of this material. The current challenge lies in translating such efficiencies to commercially feasible forms produced through industrial fabrication methods. Herein, a novel first step towards the processability of starch-perovskite inks, developed in our previous work, is investigated, by using inkjet printing technology. The tunability of the viscosity of the starch-perovskite-based inks allows the selection of suitable concentrations to be used as printable inks. After exploration of several printing parameters, thick and opaque starch-perovskite nanocomposite films were obtained, showing interesting morphological and optical properties. The results obtained in this work underline the potential and versatility of our approach, opening the possibility to explore and optimize, in the future, further large-scale deposition methods towards fully printed and stable perovskite devices.

## 1. Introduction

Inkjet printing, traditionally used in the graphics and publishing industries, has proven to be an effective digital manufacturing technique for printed solar cells [1]. In this direction, the emergence of organic–inorganic hybrid perovskite solar cells has represented a breakthrough technology, combining solution-based deposition techniques with greater efficiencies than organic solar cells [1].

However, one of the most important challenges now for perovskite-based materials is improving stability and reproducibility during fabrication, particularly by translating the high performance obtained at laboratory scale to industrial fabrication methods [2,3,4].

Among the different potential techniques for depositing perovskite-based materials and other functional layers of a solar energy device, inkjet printing is one of the most frequently used on a laboratory scale due to its versatility and selectivity in the fabrication of functional layers from solutions or suspension inks [5]. The use of this deposition technique has many advantages, such as the low-cost process, the reduced waste of the ink during deposition, the easy deposition of fine patterns at high resolution on various substrates, and above all, the scalability, enabling rapid translation of learning from small-scale, laboratory-based research into large-scale industrial roll-to-roll manufacturing [1,5,6].

The first application of inkjet printing of perovskite was reported in 2014 by Yang and co-workers [7] for the fabrication of metal-electrode-free perovskite solar cells. They used inkjet printing to deposit Methylammonium iodide (MAI) on top of a pre-deposited PbI_2_ layer, in a double step fashion. This approach led to well defined crystallinity in the Methylammonium lead tri-iodide (MAPbI_3_) thin film, reaching high performance in solar cells of power conversion efficiency (PCE) = 11.60%, open circuit voltage (*V*_oc_) = 0.95 V, short circuit current density (*J*_sc_) = 17.20 mA cm^−2^, and fill factor (FF) = 71%. Song and co-workers [8] investigated the influence of the printing table temperature of MAI and PbI_2_ on mesoporous TiO_2_, showing a strong correlation between printing table temperature and film morphology. A mild temperature (50 °C) was found to result in larger crystals with high surface coverage, obtaining a maximum PCE of 7.9%. By changing the perovskite composition and adding MACl, the morphology and device performance were further improved, achieving PCE ~12.3%, *V*_oc_ ~0.91 V, *J*_sc_ ~19.55 mA cm^−2^, and FF ~69%. Hashmi et al. reported an all-printable and ambient-processed hole transport layer (HTL) -free mesoporous perovskite solar cell. The device architecture was glass/ Fluorine-doped Tin Oxide (FTO)/Titanium dioxide (TiO_2_)/Zirconium dioxide (ZrO_2_)/MAPbI_3_/carbon, and was fabricated by infiltrating the MAPbI_3_ precursors by piezo based inkjet printing in screen printed TiO_2_, ZrO_2_, and carbon layers [9]. They have used 5-ammonium valeric acid iodide (5-AVAI) in perovskite ink as a templating agent to improve the crystalline network and charge-carrier lifetime of MAPbI_3_. This additive also prevented the clogging of inkjet nozzles by slowing-down the perovskite crystal growth. Highly reproducible and stable devices were achieved with the highest PCE of 7.83% under forward bias scan and 8.74% under reverse bias scan. Venkataraman et al. have tuned the composition of the perovskite layer for improving the performance and stability by in situ mixing of cations, Methylammonium (MA), and Formamidinium (FA) from separate ink cartridges using Red-Green-Blu (RGB) color codes of the multichannel inkjet printer [10]. They showed PCE of 11.1%, *V*_oc_ ~0.87 V, *J*_sc_ ~18.77 mA cm^−2^, FF ~68% in the p–i–n structure solar cell based on perovskite containing a MA:FA ratio of 2:1. Mathies et al. [11] introduced a vacuum-annealing step after the printed of precursor ink in one step, in order to replace the antisolvent quenching step used during spin-coating, which could not be adapted to ink-jet-printed perovskite. Three sublayers and a drop spacing of 45 μm led to a PCE of 11.3%. By following the same approach, Liang et al. [12] investigated the vacuum-assisted thermal annealing (VTA) post-treatment after the printing of perovskite, to accelerate the solvent evaporation and obtain a better morphology. Indeed, PCE of 17.04% was achieved for a small area device (0.04 cm^2^) and of 13.27% for a large area (4 cm^2^) in one-step printed deposition by using a mesoporous architecture.

The highest performance with mesoporous architecture was achieved by Li et al. [13] with a double step deposition. By exploring PbI_2_ ink precursor with various solvent compositions and using Methylammonium iodide vapor as a reaction agent to transform PbI_2_ to the MAPbI_3_, they reported a PCE of 18.63% for a small area device (0.04 cm^2^).

In this work, the printability of new inks based on perovskite and starch was investigated for the first time in view of a future development of a more scalable and cost-effective method. Very importantly, the composite perovskite:starch was proven to be more stable than the pristine perovskite. Herein, several starch-perovskite-based solutions, developed and previously deposited via spin coating [2], were investigated as inks for one-step ink-jet-printed deposition by optimizing all of the printing parameters. The best nanocomposite printed films were characterized by X-ray diffraction, UV visible absorption, morphological analyses (Optical microscope and Scanning electron microscopy), and photoluminescence measurements in view of future implementation in a full printed device.

## 2. Materials and Methods

### 2.1. Materials

Lead (II) iodide PbI_2_ ultra-dry 99.999% (metals basis) was purchased from Alfa Aesar (Haverhill, MA, USA), and Methylammonium iodide CH_3_NH_3_I (MAI) from GreatCell Solar (Queanbeyan, Australia). The corn starch used, Maizena, characterized by an A-type waxy corn structure [14], was supplied from Unilever (London, UK). Dimethyl sulfoxide anhydrous 99.9 % (DMSO) and chlorobenzene anhydrous 99.8% (CB) were purchased from Aldrich (Saint Louis, MO, USA). Poly [N,N0-bis(4-butylphenyl)-N,N0-bis(phenyl) benzidine] (poly-TPD) was from Solaris Chem Inc. (Saint-Lazare, QC, Canada). All the materials were used as received without any further purification.

### 2.2. Methods

#### 2.2.1. Poly-TPD Solution Preparation

The solution of poly-TPD (1.5 mg/mL in chlorobenzene) was prepared by stirring at 40 °C for 2 h.

#### 2.2.2. Starch-Perovskite Solution Preparation

The perovskite precursor solution containing an equimolar precursor stoichiometry, i.e., MAI:PbI_2_ = 1:1, was prepared by mixing MAI with PbI_2_ in DMSO with varying precursor weight concentrations in DMSO, i.e., 10–20–30 wt%, as reported in Table 1. The solutions were stirred on a hot plate at 80 °C for 30 min. After precursor solubilization, different starch/precursor ratios were added to each solution, i.e., 1, 5, 10, and 15 wt%, and were stirred at 80 °C for 5 h in order to obtain a clear solution after starch solubilization. The identification name (ID) of each sample with the correspondent precursors and starch concentrations are reported in Table 1.

#### 2.2.3. Rheological Characterization of Perovskite Precursors Solutions

The viscosity of the perovskite precursor solutions by varying the precursor concentrations in DMSO from 5 to 30 wt% and with a starch/precursor ratio from 1 to 15 wt%, was carried out in a strain controlled rheometer (Malvern Kinexus Pro+, Malvern, UK) equipped by parallel plate geometry (radius = 12.5 mm) in steady state mode with a shear rate ranging from 1–1000 s^−1^ at 23 °C. All the liquid formulations were tested after stirring at 80 °C for 5 h and cooled to ambient temperature. This was in order to better understand the influence of precursor and starch concentrations on the viscosity of the as-prepared perovskite solutions before deposition. The rheological experiments were repeated three times to check the repeatability of the results.

#### 2.2.4. Surface Tension

The surface tension of the inks was measured by Krüss DSA100 (Bristol, UK) using the pendant drop method.

#### 2.2.5. Inkjet Printing Process

Patterns of 5 mm × 5 mm with various print resolutions were designed using the open source software, GIMP (The General Image Manipulation Program, Spencer Kimball, Peter Mattis, 2.10.8 version, Berkeley, CA, USA). The files were saved as bitmap (.bmp) image file format and opened with Dimatix drop manager (Fujifilm, Santa Clara, CA, USA) in order to be loaded by the printer software.

The printing frequency was set at 5.0 kHz and a customized waveform was used. The voltage and the temperature of the cartridge were optimized based on the ink explored. The printing process was carried out in air with a humidity of 20–30%. The temperature of the substrate was optimized during the process through print platform heating. The diameters of the printed drops were analyzed to optimize the drop spacing of each explored ink.

The substrate was prepared by washing the glass slide with isopropanol. Then, the poly-TPD solution (1.5 mg/mL in clorobenzene) was printed at room temperature by using the same customized waveform with a drop spacing of 60 μm (distance between droplets center to center) and a voltage of 23 V. Printed poly-TPD was annealed in air at 140 °C for 30 min, followed by UV treatment of 30 min in order to improve the wettability, as already demonstrated in our previous paper [2].

#### 2.2.6. Morphological Characterization

The surface morphology of the single droplets printed and of the printed starch-perovskite composite samples was analyzed by an optical microscope (Nikon Eclipse–LV100ND, Tokyo, Japan). Scanning electron microscopy (SEM) images of the printed starch-perovskite composite samples, deposited on a glass/printed poly-TPD substrate, were collected by using Carl Zeiss Auriga 40 Crossbeam instrument (Oberkochen, Germany), in high vacuum and high-resolution acquisition mode, equipped with Gemini column and an integrated high efficiency in-lens detector. The applied acceleration voltage was 5 kV.

#### 2.2.7. XRD Measurements

Wide-angle X-ray diffraction (XRD) patterns were obtained in reflection geometry by an automated Bruker D8 Advance diffractometer (Billerica, MA, USA), equipped with nickel-filtered Cu Kα radiation (1.5418 Å) as the X-ray source that was operated at 35 kV and 40 mA.

#### 2.2.8. UV-vis Absorption

UV-visible optical absorption spectra were recorded on Varian Cary 500 spectrophotometer (Agilent, Santa Clara, CA, USA) in the 200–800 nm wavelength range at room temperature.

#### 2.2.9. Photoluminescence (PL) Experiments

Steady state and time resolved photoluminescence (PL) was measured by an Edinburgh FLS920 spectrometer (Livingstone, UK) equipped with a Peltier-cooled Hamamatsu R928 photomultiplier tube (185–850 nm). An Edinburgh Xe900 450 W Xenon arc lamp (Livingstone, UK) was used as exciting light source. Corrected spectra were obtained via a calibration curve supplied with the instrument (lamp power in the steady state PL experiments ~0.6 mW cm^−2^, spot area 0.5 cm^2^). Emission lifetimes were determined with the single photon counting technique by means of the same Edinburgh FLS980 spectrometer (Livingstone, UK), using a laser diode as excitation source (1 MHz, λ_exc_ = 635 nm, 67 ps pulse width, and about 30 ps time resolution after deconvolution) and a Hamamatsu MCP R3809U-50 (time resolution 20 ps) as detector (laser power in the time resolution photoluminescence (TRPL) experiment ~1.6 W cm^–2^, spot area 0.3 mm^2^) [15].

## 3. Results and Discussion

The printability of the starch-perovskite-based inks [2] was explored with the aim to evaluate the possibility to deposit them through large scale methods. The printability of a solution can be defined by the Z parameter that is directly dependent on viscosity, surface tension, and density of the ink [16,17,18]. Therefore, these properties were, firstly, evaluated in order to select the solutions more suitable for ink jet printing technology.

**Figure 1:**
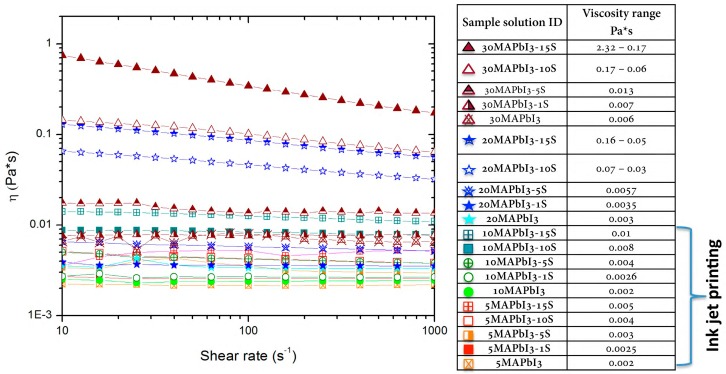
Rheological characterization of the perovskite-starch-based solutions investigated by varying the precursors and starch concentrations.

Figure 1 showed the steady state viscosity of the different solutions investigated, including a wide range of perovskite precursor concentrations from 5 to 30 wt% and starch/precursor concentrations from 0 to 15 wt%. All the perovskite precursor formulations, without starch, showed a Newtonian behavior with almost constant viscosity ranges from ~0.002 to ~0.007 Pa s by increasing the precursors content. By increasing the starch content, at lower perovskite precursor concentrations, i.e., 5 and 10 wt%, the solutions still showed a Newtonian behavior, with an increased viscosity up to ~0.01 Pa s. By increasing the perovskite precursors ratio to 20 and 30 wt%, a pseudoplastic behavior was observed in the presence of starch content higher than 5 wt%.

The starch used for the solutions was widely characterized in our previous work [14], as well as its role as a rheological modifier [2], with the interesting discovery that the gelatinization process of the starch into DMSO together with the interaction between the OH groups of the starch and the perovskite precursors highly influences the rheological behavior of the solutions. Interestingly, the results of the rheological characterization showed that by tuning the concentration of the precursors and of the starch, it is possible to obtain a solution with rheological properties suitable to the deposition technology used. By decreasing the perovskite precursor ratio down to 5–10 wt%, we were able to move from spin coating to ink jet printing of perovskite-starch deposition. Together with the viscosity, the surface tension and the density of the inks are key parameters to investigate the “ejectability” of a solution. In general, values between 20–40 mN/m are considered more suitable for ink jet printing processes, lower than the surface tension values reported in Table 2 for the solutions with varying perovskite precursors, from 5 to 10 wt%, and starch content from 0 to 10 wt%. The respective densities, calculated by weighing a known volume of each solution, were reported in Table 2, together with the *Z* value calculated by Equation (1).

*Z* = (*ρaγ*)^1/2^/*η* (1)

where *η* is the dynamic viscosity, *ρ* is the density, *γ* is the surface tension, *a* is the characteristic length (usually the nozzle diameter) of the ink.

The solution containing the 5 wt% of perovskite precursor in solvent showed a surface tension of 44.24 ± 0.15 mN/m, close to the solvent used for the solution, DMSO (45.25 ± 0.47 mN/m). In the presence of starch, as well as with a higher precursor concentration, the surface tension increases up to about 53 mN/m. As reported in Table 2, *Z* values decreased by increasing the starch content, driven by the increased viscosity, and were much lower for the ink containing 10 wt% of precursors, showing a good influence of the presence of the starch on the ink ejectability calculated from *Z*. Indeed, *Z* values between 1 and 10 are more suitable for ink jet printing technology, even though the value of a printable *Z* number is still under investigation [19,20,21].

**Figure 2:**
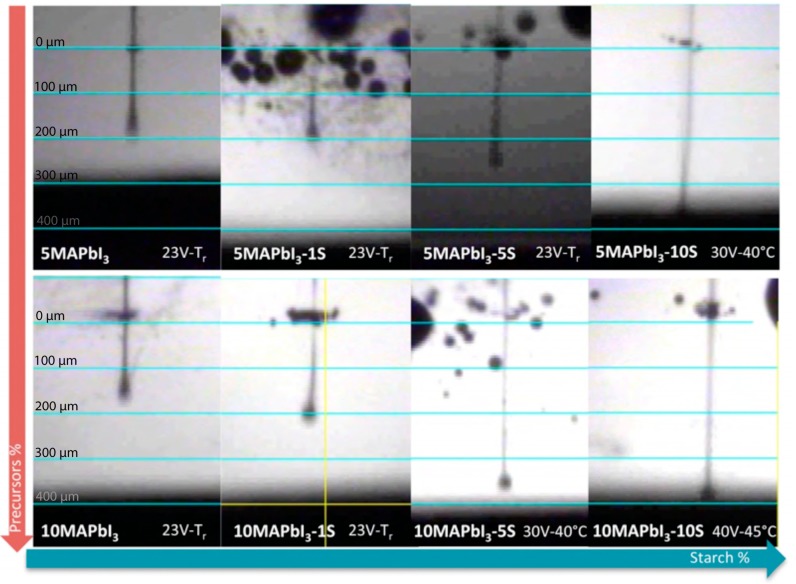
Droplets ejected from the nozzle for the different inks explored.

Subsequently, the ejectability of the inks was experimentally investigated by applying several customized waveforms to the nozzle of the cartridge and by optimizing the voltage and the temperature applied to obtain a drop ejected from the nozzle, as shown in Figure 2.

After optimization of the previous parameters, interesting results were obtained for most of the inks reported in Table 1, by setting the voltage of the piezoelectric driven print-heads at each nozzle at 23 V and the temperature of the print-head at 23 °C. On the other hand, for the formulations containing higher starch concentration, 5MAPbI_3_-10S, 10MAPbI_3_-5S, and 10MAPbI_3_-10S, higher voltage and temperature were necessary to allow the ejection of the ink from the nozzle, i.e., 30 V and 40 °C, and 40 V and 45 °C, respectively. This latter result was probably due to the combination of higher surface tension and viscosity, despite the lower *Z* parameter calculated. It is interesting to observe that the shape and the length of the drop were highly influenced by the concentration of starch. In detail, increasing starch molecular concentration lead to a longer drop tail due to greater long chain content, typical of polymers with high molecular weight-based solutions [22].

**Figure 3:**
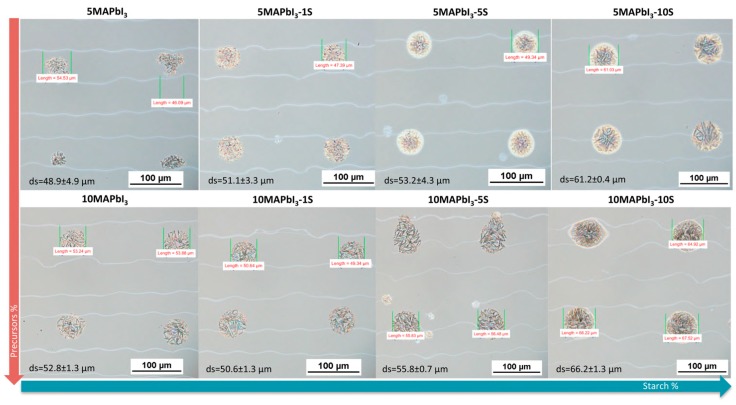
Single droplets of the inks deposited on printed and annealed poly-TPD by varying perovskite precursors and starch concentrations.

Figure 3 shows single droplets of the perovskite inks deposited on printed and annealed poly-TPD by varying perovskite precursors and starch concentrations, before thermal treatment. It is evident that the size of the drops increases from about 48.9 ± 4.9 μm for 5 wt% and 52.8 ± 1.3 μm for 10 wt%, in absence of starch, up to 61.2 ± 0.4 μm and 66.2 ± 1.3 μm, respectively, after adding 10 wt% of the biopolymer. Moreover, the presence of the starch allows for a more compact and homogeneous drop on the substrate to be obtained, which is a key aspect in order to realize a good photoactive film.

From droplet to pattern printing, there are several aspects to consider; first, the choice of a proper drop space, in order to have the appropriate overlapping of the droplets, which is essential to obtain a continuous pattern. Hence, drop size is a crucial parameter and depends on the ink, wettability of the substrate, and surface temperature as well. As the diameter of the print head nozzle is fixed, the droplet overlap is controlled by increasing or decreasing the resolution of the image (dpi) of the pattern [23].

**Figure 4:**
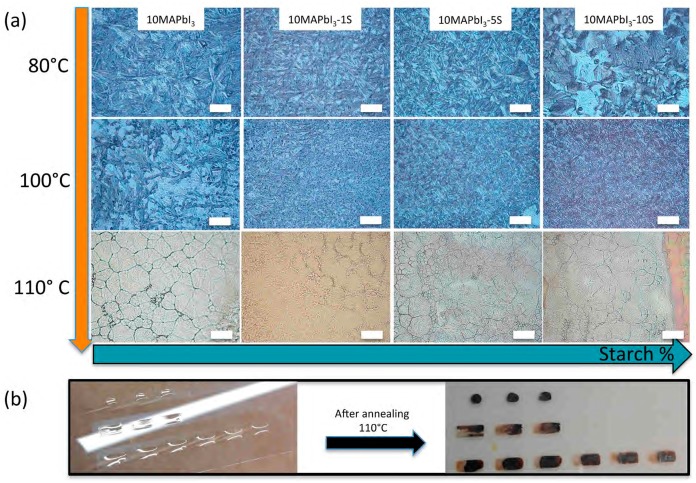
Optical microscope images of different precursor perovskite solution printed patterns annealed at different temperatures (scale bar 100 μm) (**a**), and “shift” of the drops observed after thermal annealing at 110 °C for different starch concentrations (**b**).

By considering the average drop size of the different inks tested, a drop space of 30 μm was firstly selected to be used for printing continuous patterns. In addition, several annealing temperatures, from 80 to 110 °C, were explored for printed perovskite precursor films and shown in Figure 4a.

All of the films annealed at 80 and 100 °C resulted in a needle-like morphology organized in smaller islands, by increasing the temperature and the starch content (at fixed temperature). After annealing at 110 °C, a more compact morphology was observed, irrespective of the starch content, suggesting that the rate of the solvent evaporation is a key point during perovskite film formation; by increasing the annealing temperature, then the rate of DMSO evaporation, a better perovskite grain morphology could be obtained. However, a “shift” of the drops was observed during thermal annealing, leading to a non-homogeneous perovskite film, as shown in the pictures in Figure 4b, which was more evident as the starch content decreased.

**Figure 5:**
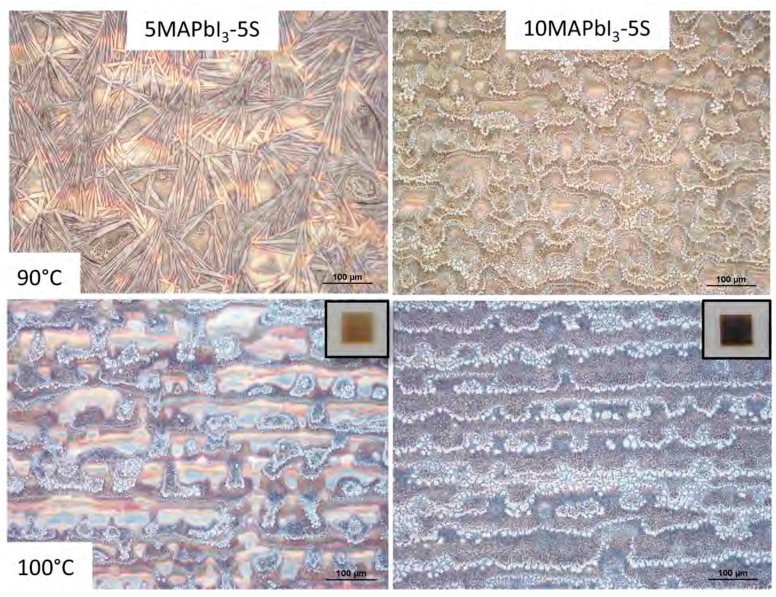
Optical images of 5MAPbI_3_-5S and 10MAPbI_3_-5S samples printed at 90° and 100 °C, and photo of the 5 × 5 mm^2^ printed pattern in the inset.

Furthermore, with the aim to overcome the crystallization issue by increasing the solvent evaporation rate, and to better control the uniformity of the printed pattern, the perovskite precursor-based inks containing 5 and 10 wt% of precursors and 5 wt% of starch were printed on a hot substrate at 90 and 100 °C. As shown in the optical images reported in Figure 5, for both of the concentrations explored, a higher temperature of the substrate enabled a morphology characterized by perovskite grains, whereas a needles-like structure was observed by depositing the 5 wt% of starch perovskite at 90 °C. Therefore, 100 °C was selected as the best printing temperature. Moreover, a well-defined 5 × 5 mm^2^ printed pattern was obtained, as shown in the inset of Figure 5.

**Figure 6:**
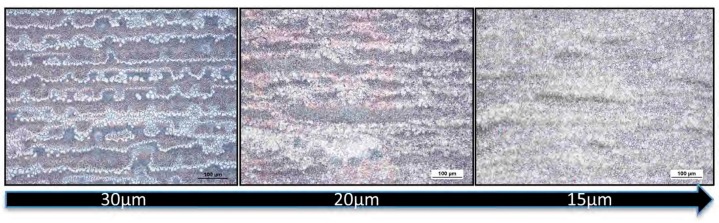
The 10MAPbI_3_-5S samples printed at 100 °C by using a drop spacing of 30, 20, and 15 µm.

With the aim to improve the coverage of the substrate, the drop space was decreased from 30 to 15 µm, leading to a more compact and homogeneous film, as shown in Figure 6 for 10MAPbI_3_-5S perovskite ink.

Therefore, in the following experiment, 5MAPbI_3_-5S and 10MAPbI_3_-5S were printed according to the optimized parameters and the respective films were accurately characterized and compared.

**Figure 7:**
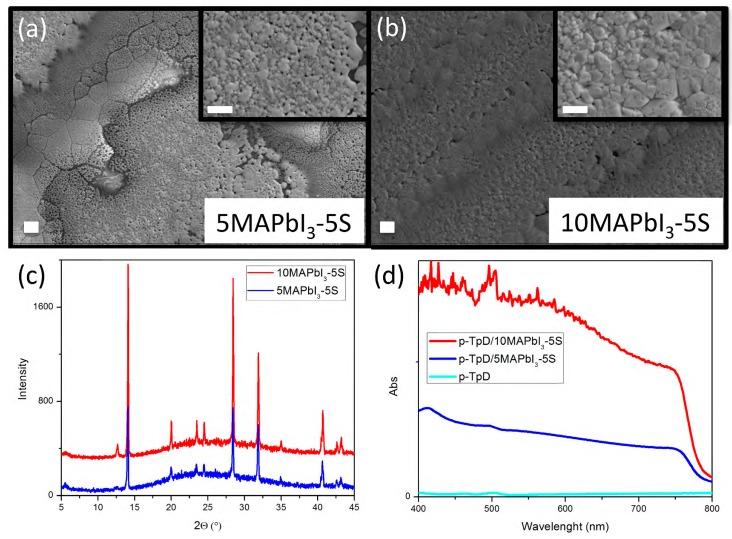
Scanning electron microscopy (SEM) images (**a**,**b**), X-ray diffraction (XRD) spectra (**c**), and UV-visible absorption (**d**) of 5MAPbI_3_-5S and 10MAPbI_3_-5S printed pattern film on glass/printed poly-TPD substrate. Scale bars 10 μm.

The SEM images in Figure 7a,b show a morphology characterized by large grains of about 5 µm in both samples. However, slightly larger and more compact grains were observed for 10MAPbI_3_-5S, combined with a smaller quantity of gaps than 5Mapi-5S film. The X-ray diffraction patterns on 5MAPbI_3_-5S and 10MAPbI_3_-5S printed film in Figure 7c display a strong peak at 14.2° of the MAPbI_3_ crystal. However, the presence of a small peak at 12.7°, in both of the samples, suggested the presence of unreacted PbI_2_ in the film, maybe due to the deposition conditions (in air, in presence of humidity). The characteristic absorption spectra of perovskite were shown for both of the concentrations explored in Figure 7d, with higher absorption intensity by increasing the precursor concentration due to the increasing thickness of the film from about 1 µm (5MAPbI_3_-5S) to 1.7 µm (10MAPbI_3_-5S). It is interesting to observe that thicker and more opaque films were achieved by inkjet depositing perovskite inks at low precursor concentrations of 5 and 10 wt%, when compared to conventional spin-coating [2]. Moreover, despite the high thickness, printed perovskite films are highly converted, as demonstrated by XRD patterns, which did not evidence the presence of peaks related to MAI-DMSO-PbI_2_ intermediate complex (6.6°, 7.2°, and 9.2°) [24].

**Figure 8:**
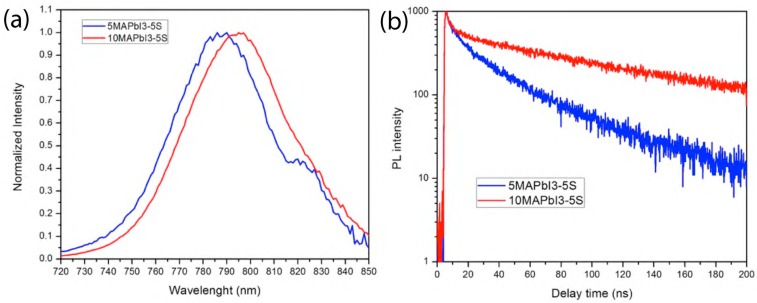
Photoluminescence (PL) spectra (**a**) and PL lifetime decay (**b**) of 5MAPbI_3_-5S and 10MAPbI_3_-5S printed pattern film.

Aiming at future optoelectronic applications, the optical properties of the printed films were investigated by steady-state PL measurements, as shown in Figure 8. The intense PL emission band of 10MAPbI_3_-5S peaks at 795 nm, and it is slightly blue, shifting to 790 nm for 5MAPbI_3_-5S. The emission band position and intensities are in line with what was previously reported for these composites, and suggests good optoelectronic properties for the perovskite component of the ink [25].

The time-resolved PL experiment (Figure 8b) further supports the claim of good optoelectronic properties, as the lifetimes are reasonably long (9, 52 ns for 5MAPbI_3_-5S; and 5, 124 ns for 10MAPbI_3_-5S) and suggest reduced parasite traps mediated the carrier’s recombination. The role of the starch has already been proven to positively affect the optoelectronic properties of perovskite materials. Herein, the increase of the lifetime associated to a higher precursor concentration suggests better optoelectronic properties for 10MAPbI_3_-5S film, which could be due to an increase of the perovksite grain dimensions.

## 4. Conclusions

In this work, the possibility to print the starch-perovskite inks, developed in our previous work, was explored by using ink jet printing as a first step towards a more scalable deposition process of perovskite-based devices. The tunability of the viscosity of the starch-perovskite-based inks allowed us to select the suitable concentration to be used as printable inks. Through the investigation of the printing parameters, such as waveform, voltage, and temperature of piezoelectric driven print heads from one side, and drop space, substrate temperature from the other, printed perovskite films with good morphological, crystalline, and optical properties were realized. These results represent the first demonstration of the versatility of the perovskite deposition approach developed during this work, and open the possibility for further investigations towards upscaling of perovskite deposition.

## Figures and Tables

**Table 1:** Samples ID and compositions.

Sample ID	MAPbI_3_/DMSO (wt%)	Starch/MAPbI_3_ (wt%)
5MAPbI_3_	5	0
5MAPbI_3_-1S	5	1
5MAPbI_3_-5S	5	5
5MAPbI_3_-10S	5	10
5MAPbI_3_-15S	5	15
10MAPbI_3_	10	0
10MAPbI_3_-1S	10	1
10MAPbI_3_-5S	10	5
10MAPbI_3_-10S	10	10
10MAPbI_3_-15S	10	15
10MAPbI_3_-20S	10	20
20MAPbI_3_	20	0
20MAPbI_3_-1S	20	1
20MAPbI_3_-5S	20	5
20MAPbI_3_-10S	20	10
20MAPbI_3_-15S	20	15
20MAPbI_3_-20S	20	20
30MAPbI_3_	30	0
30MAPbI_3_-1S	30	1
30MAPbI_3_-5S	30	5
30MAPbI_3_-10S	30	10
30MAPbI_3_-15S	30	15

**Table 2:** Density, surface tension, viscosity, and *Z* parameter of the inks explored.

Ink	Density (g/mL)	Surface Tension (mN/m)	Viscosity at 23 °C (mPa s)	*Z*
5MAPbI_3_	1.095	44.24 ± 0.15	2	15.94
5MAPbI_3_-5S	1.369	53.34 ± 0.15	3	13.05
5MAPbI_3_-10S	1.365	53.15 ± 0.27	4	9.75
10MAPbI_3_	1.375	53.39 ± 0.32	2	19.63
10MAPbI_3_-5S	1.392	53.32 ± 0.16	4	9.86
10MAPbI_3_-10S	1.410	53.30 ± 0.33	8	4.96

## References

[1] Peng X., Yuan J., Shen S., Gao M., Chesman A.S.R., Yin H., Cheng J., Zhang Q., Angmo D. (2017). Perovskite and organic solar cells fabricated by inkjet printing: progress and prospects. Adv. Funct. Mater..

[2] Giuri A., Masi S., Listorti A., Gigli G., Colella S., Corcione C.E., Rizzo A. (2018). Polymeric rheology modifier allows single-step coating of perovskite ink for highly efficient and stable solar cells. Nano Energy.

[3] Ding B., Li Y., Huang S.-Y., Chu Q.-Q., Li C.-X., Li C.-J., Yang G.-J. (2017). Material nucleation/growth competition tuning towards highly reproducible planar perovskite solar cells with efficiency exceeding 20%. J. Mater. Chem. A.

[4] Wang Z.-K., Gong X., Li M., Hu Y., Wang J.-M., Ma H., Liao L.S. (2016). Induced crystallization of perovskites by a perylene underlayer for high-performance solar cells. ACS Nano.

[5] Van Franeker J.J., Voorthuijzen W., Gorter H., Hendriks K.H., Janssen R.A., Hadipour A., Andriessen R., Galagan Y. (2013). All-solution-processed organic solar cells with conventional architecture. Sol. Mater. Sol. Cells.

[6] Singh M., Haverinen H.M., Dhagat P., Jabbour G.E. (2010). Inkjet printing—Process and its applications. Adv. Mater..

[7] Wei Z., Chen H., Yan K., Yang S. (2014). Inkjet printing and instant chemical transformation of a CH_3_NH_3_PbI_3_/nanocarbon electrode and interface for planar perovskite solar cells. Angew. Chem. Int. Ed..

[8] Li S.-G., Jiang K.-J., Su M.-J., Cui X.-P., Huang J.-H., Zhang Q.-Q., Zhou X.-Q., Yang L.-M., Song Y.-L. (2015). Inkjet printing of CH_3_NH_3_PbI_3_ on a mesoscopic TiO_2_ film for highly efficient perovskite solar cells. J. Mater. Chem. A.

[9] Hashmi S.G., Martineau D., Li X., Ozkan M., Tiihonen A., Dar M.I., Sarikka T., Zakeeruddin S.M., Paltakari J., Lund P.D. (2017). Air processed inkjet infiltrated carbon based printed perovskite solar cells with high stability and reproducibility. Adv. Mater. Technol..

[10] Bag M., Jiang Z., Renna L.A., Jeong S.P., Rotello V.M., Venkataraman D. (2016). Rapid combinatorial screening of inkjet-printed alkyl-ammonium cations in perovskite solar cells. Mater. Lett..

[11] Mathies F., Abzieher T., Hochstuhl A., Glaser K., Colsmann A., Paetzold U.W., Hernandez-Sosa G., Lemmer U., Quintilla A. (2016). Multipass inkjet printed planar methylammonium lead iodide perovskite solar cells. J. Mater. Chem. A.

[12] Liang C., Li P., Gu H., Zhang Y., Li F., Song Y., Shao G., Mathews N., Xing G. (2018). One-Step Inkjet Printed Perovskite in Air for Efficient Light Harvesting. Sol. RRL..

[13] Li P., Liang C., Bao B., Li Y., Hu X., Wang Y., Zhang Y., Li F., Shao G., Song Y. (2018). Inkjet manipulated homogeneous large size perovskite grains for efficient and large-area perovskite solar cells. Nano Energy.

[14] Giuri A., Colella S., Listorti A., Rizzo A., Corcione C.E. (2018). Biodegradable extruded thermoplastic maize starch for outdoor applications. J. Therm. Anal..

[15] Masi S., Aiello F., Listorti A., Balzano F., Altamura D., Giannini C., Caliandro R., Uccello-Barretta G., Rizzo A., Colella S. (2018). Connecting the solution chemistry of PbI_2_ and MAI: A cyclodextrin-based supramolecular approach to the formation of hybrid halide perovskites. Chem. Sci..

[16] Zhang F., Tuck C., Hague R., He Y., Saleh E., Li Y., Sturgess C., Wildman R. (2016). Inkjet printing of polyimide insulators for the 3D printing of dielectric materials for microelectronic applications. J. Appl. Polym. Sci..

[17] Gheno A., Huang Y., Bouclé J., Ratier B., Rolland A., Even J., Vedraine S. (2018). Toward Highly Efficient Inkjet-Printed Perovskite Solar Cells Fully Processed Under Ambient Conditions and at Low Temperature. Sol. RRL.

[18] Zhumekenov A.A., Burlakov V.M., Saidaminov M.I., Alofi A., Haque M.A., Turedi B., Davaasuren B., Dursun I., Cho N., El-Zohry A.M. (2017). The Role of Surface Tension in the Crystallization of Metal Halide Perovskites. ACS Lett..

[19] Tai J., Gan H.Y., Liang Y.N., Lok B.K. Control of Droplet Formation in Inkjet Printing Using Ohnesorge Number Category: Materials and Processes. Presented at the 2008 10th Electronics Packaging Technology Conference (EPTC 2008).

[20] Jang D., Kim D., Moon J. (2009). Influence of Fluid Physical Properties on Ink-Jet Printability. Langmuir.

[21] Saleh E., Woolliams P., Clarke B., Gregory A., Greedy S., Smartt C., Wildman R., Ashcroft I., Hague R., Dickens P. (2017). 3D inkjet-printed UV-curable inks for multi-functional electromagnetic applications. Addit. Manuf..

[22] De Gans B., Duineveld P.C., Schubert U.S. (2004). Inkjet printing of polymers: State of the art and future developments. Adv. Mater..

[23] Vaithilingam J., Simonelli M., Saleh E., Senin N., Wildman R.D., Leach R.K., Tuck C.J., Hague R.J. (2017). Combined Inkjet Printing and Infra-red Sintering of Silver Nanoparticles using a Swathe-by-swathe and Layer-by-layer approach for 3-dimensional Structures. ACS Appl. Mater. Interfaces.

[24] Jeon N.J., Noh J.H., Kim Y.C., Yang W.S., Ryu S., Seok S.I. (2014). Solvent engineering for high-performance inorganic–organic hybrid perovskite solar cells. Nat. Mater..

[25] Giuri A., Yuan Z., Miao Y., Wang J., Gao F., Sestu N., Saba M., Bongiovanni G., Colella S., Corcione C.E. (2018). Ultra-Bright Near-Infrared Perovskite Light-Emitting Diodes with Reduced Efficiency Roll-off. Sci. Rep..

